# Two new species of *Pseudancistrus* (Siluriformes, Loricariidae) from the Amazon basin, northern Brazil

**DOI:** 10.3897/zookeys.482.6909

**Published:** 2015-02-11

**Authors:** Gabriel S. C. Silva, Fábio F. Roxo, Claudio Oliveira

**Affiliations:** 1 Laboratório de Biologia e Genética de Peixes, Departamento de Morfologia, IB-UNESP, Campus de Botucatu, 18618-970, SP, Brazil

**Keywords:** Ancistrini, Neotropical fish, freshwater, Brazilian Shield, taxonomy

## Abstract

Two new species of *Pseudancistrus*, a genus diagnosed by non-evertible cheek plates and hypertrophied odontodes along the snout margin, are described from two drainages of the Brazilian Shield: *Pseudancistrus kayabi* from the rio Teles Pires (rio Tapajós basin) and *Pseudancistrus asurini* from the rio Xingu. The new species are distinguished from congeners (*Pseudancistrus barbatus*, *Pseudancistrus corantijniensis*, *Pseudancistrus depressus*, *Pseudancistrus nigrescens*, *Pseudancistrus reus*, and *Pseudancistrus zawadzkii*) by the coloration pattern. *Pseudancistrus kayabi* has dark bars on the dorsal and caudal fins which are similar to that of *Pseudancistrus reus* from the Caroní River, Venezuela. *Pseudancistrus asurini* is unique among *Pseudancistrus* in having whitish tips of the dorsal and caudal fins in juveniles to medium-sized adults.

## Introduction

With 892 species, the suckermouth armoured catfish family Loricariidae is the fifth most species-rich family of vertebrates and one of the most species-rich groups among Neotropical fishes (Eschmeyer and Fong 2014). The loricariids are easily distinguished by having a ventral oral disk, the body covered with ossified dermal plates, and the presence of small external teeth known as odontodes. Within this family, all species that have highly evertible clusters of cheek odontodes are placed within the subfamily Hypostominae, the tribe Ancistrini (Armbruster 2004a, 2008). Morphology-based studies by Armbruster (2004a, 2008) showed Ancistrini as a monophyletic group; however, recent molecular studies supported the conclusion that the tribe was polyphyletic (e.g. Covain and Fish-Muller 2012; Lujan et al. 2015). Ancistrini was redefined by Lujan et al. (2015) and currently includes only ten valid genera but stays the second most genus-rich of the nine tribe-level clades of Hypostominae.

*Pseudancistrus* Bleeker, 1862 was known to contain 15 valid species (Eschmeyer and Fong 2014) but recent publications (e.g. Chambrier and Montoya-Burgos 2008; Covain and Fisch-Muller 2012; Silva et al. 2014; Lujan et al. 2015) revealed that the genus is not monophyletic and that the type species, *Pseudancistrus barbatus* (Valenciennes, 1840), is closely related only with four species known as the *Pseudancistrus barbatus* species group: *Pseudancistrus corantijniensis* de Chambrier & Montoya-Burgos, 2008, *Pseudancistrus depressus* (Günther, 1868), *Pseudancistrus nigrescens* Eigenmann, 1912, and *Pseudancistrus zawadzkii* Silva, Roxo, Britzke & Oliveira, 2014, the latter being the only species described to date from rivers flowing from the Brazilian Shield into the Amazon. Other species not included in these works were considered to possibly belong to *Pseudancistrus*: *Pseudancistrus guentheri* (Regan, 1904) *Pseudancistrus kwinti* Willink, Mol & Chernoff, 2010 (Covain and Fisch-Muller 2012), and *Pseudancistrus reus* Armbruster & Taphorn, 2008 (Lujan et al. 2015). This last work retained *Pseudancistrus reus* as the only species belonging to this group from the eastern Orinoco basin. The genus *Pseudancistrus* is diagnosed by a combination of characters state as follows: a depressed body, hypertrophied odontodes along the lateral margin of the snout (regardless of either sex or season), and hypertrophied cheek odontodes which are evertible to less than 45° from the body (Lujan et al. 2015).

Recently, an examination of the fish collections at the LBP (Laboratório de Biologia e Genética de Peixes de Botucatu) and MZUSP (Museu de Zoologia da Universidade de São Paulo) revealed the existence of two undescribed species of *Pseudancistrus* from the rio Xingu (the first species of *Pseudancistrus* for this basin) and the rio Teles Pires (the second species of *Pseudancistrus* for rio Tapajós basin), both of which are tributaries of the Amazon basin draining the Brazilian Shield. In the present paper these two new species are described.

## Material and methods

After capture, fishes were anesthetized using 1% benzocaine in water, fixed in 10% formaldehyde, and preserved in 70% ethanol. Vouchers and tissues were deposited in the collection of AUM (Auburn University Natural History Museum, Auburn, USA), LBP (Laboratório de Biologia e Genética de Peixes, Botucatu, Brazil), and MZUSP (Museu de Zoologia da Universidade de São Paulo, São Paulo, Brazil). Measurements and counts were taken from the left side. Body plate follows Schaefer (1997) and measurements were taken point to point to the nearest 0.1 mm using digital calipers on left side of specimens following Armbruster (2003). Morphometrics are given as percentages of standard length (SL), except for subunits of the head region that are expressed as percentages of head length (HL). Dorsal-fin ray counts include the spinelet as the first unbranched ray. Zoological nomenclature follows the International Code of Zoological Nomenclature (International Commission on Zoological Nomenclature 1999).

## Results

### Pseudancistrus kayabi sp. n.

Taxon classificationAnimaliaSiluriformesLoricariidae

http://zoobank.org/F8B055A4-C576-4FC5-B0CF-8021F0B7DD93

Figure 1 , Table 1

#### Holotype.

MZUSP 116322, male, 88.4 mm SL. Brazil: Mato Grosso State: municipality of Itaúba: rio Teles Pires (Tapajós River basin), 10°58'30"S, 55°44'03"W, 01 October 2007, JLO Birindelli, P Hollanda-Carvalho.

#### Paratypes.

All from Brazil: Mato Grosso State: rio Teles Pires (Tapajós River basin): Amazon basin. AUM 65641 2, 74.5−80.3 mm SL, municipality of Itaúba, 11°03'44"S, 55°19'08"W, 26 September 2007, JLO Birindelli, P Hollanda-Carvalho. LBP 19552, 2, 79.1−87.1 mm SL, municipality of Itaúba, 11°03'44"S, 55°19'08"W, 26 September 2007, JLO Birindelli, P Hollanda-Carvalho. MZUSP 95851, 1, 60.9 mm SL, collected with holotype. MZUSP 95912, 54, 27.1−86.5 mm SL, municipality of Itaúba, 11°03'44"S, 55°19'08"W, 26 September 2007, JLO Birindelli, P Hollanda-Carvalho. MZUSP 96157, 34, 29.5−85.8 mm SL, municipality of Paranaíta, 09°26'58"S, 56°29'19"W, 28 September 2007, LMS Souza, AL Netto-Ferreira.

#### Diagnosis.

*Pseudancistrus kayabi* differs from all congeners except *Pseudancistrus reus* by having caudal and dorsal fins with dark bars (vs. with white spots in caudal and dorsal fins). Also, the new species differs from all *Pseudancistrus* except *Pseudancistrus nigrescens* by having a dark brown body with whitish spots that fade along the posterior portion of the dorsal fin and forming mottled pattern (vs. either dark brown with conspicuous rounded spots not covering more than one plate in *Pseudancistrus barbatus*, *Pseudancistrus corantijniensis*, *Pseudancistrus depressus*, *Pseudancistrus asurini*, and *Pseudancistrus zawadzkii* or with dark brown bars in *Pseudancistrus reus*). It further differs from *Pseudancistrus barbatus* and *Pseudancistrus depressus* by having the snout with yellowish hypertrophied odontodes (vs. reddish-brown odontodes) (see Fig. 3 in De Chambrier and Montoya-Burgos 2008 for comparison). In addition, *Pseudancistrus kayabi* is distinguished by having a shorter pectoral spine, 22−30% SL (vs. 29−34% in *Pseudancistrus nigrescens*, 31−33% in *Pseudancistrus zawadzkii*, and 30−34% in *Pseudancistrus barbatus*); a shorter dorsal-fin base, 20−28% SL (vs. 28−29% in *Pseudancistrus nigrescens*, 29−31% in *Pseudancistrus zawadzkii*, and 28−31% in *Pseudancistrus barbatus*); a greater internares width, 13−19% HL (vs. 10.5−12.9% in *Pseudancistrus nigrescens*); head depth, 60−66% HL, greater than in *Pseudancistrus nigrescens* (56−57%) and in *Pseudancistrus barbatus* (41−53%) but smaller than in *Pseudancistrus zawadzkii* (67−73%); and a greater adipose-anal distance (17−25% SL vs. 15−17% in *Pseudancistrus nigrescens* and 12−15% in *Pseudancistrus barbatus*).

**Figure 1.**
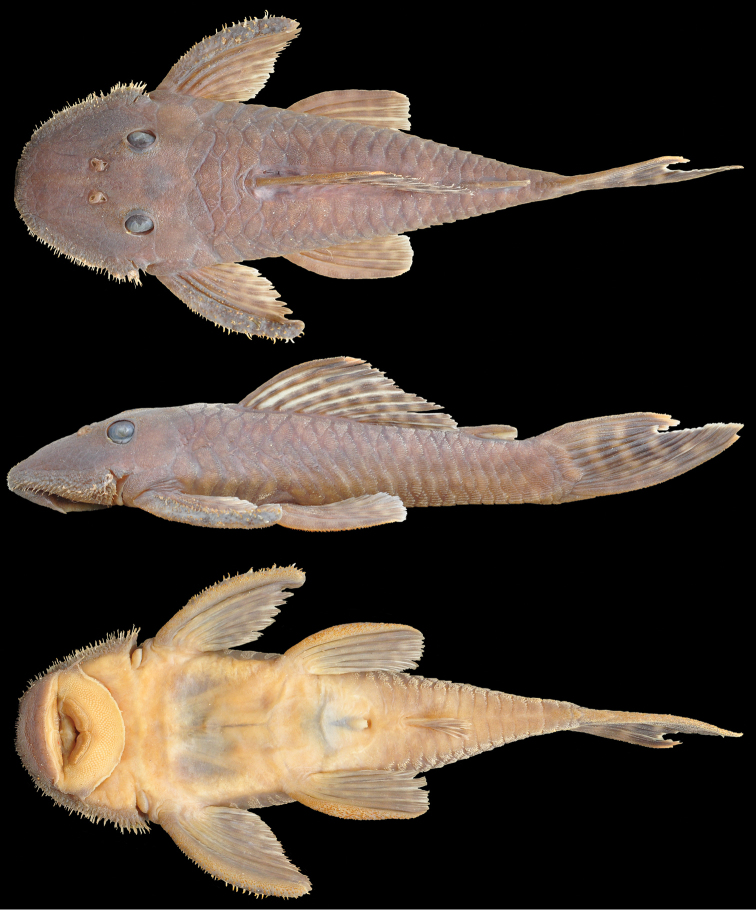
*Pseudancistrus kayabi*, holotype, MZUSP 116322, male 88.4 mm SL, from rio Teles Pires (Amazon basin), municipality of Itaúba, Mato Grosso State, Brazil.

#### Description.

Morphometric data is presented in Table 1. In lateral view, dorsal profile convex from snout tip to dorsal-fin origin; straight, gradually descending from dorsal-fin origin to posterior insertion of adipose fin; straight, steeply ascending to insertion of caudal-fin; ventral profile flat from snout tip to anal-fin origin; shallowly concave from anal-fin insertion to lower caudal-fin spine; greatest body depth at dorsal-fin origin. In dorsal view, greatest body width across cleithral region; snout broadly elliptical; body progressively narrow from opercular region to caudal fin. Cross-section of body between pectoral and pelvic fins rounded dorsally and flattened ventrally; cross-section of caudal peduncle ellipsoid.

Body almost entirely covered with plates except on ventral portions of head, abdomen, and dorsal-fin base. Five lateral rows of dermal plates, dorsal plates 21−22, lateral mid-dorsal plates 21−22, lateral median plates 22−23, lateral mid-ventral plates 21−22, lateral ventral plates 19−20. Three predorsal plates; eight plates below dorsal-fin base; four plates between dorsal fin and adipose fin; five rows of plates on caudal peduncle. Dorsal spinelet present.

**Table 1.** Morphometric data for *Pseudancistrus kayabi* and *Pseudancistrus asurini*. SD = standard deviation.

	*Pseudancistrus kayabi* n = 21				*Pseudancistrus asurini* n = 21			
	Holotype	Range	Mean	SD	Holotype	Range	Mean	SD
**SL**	88.4	61.5−87.7	78.5	7.2	195.8	195.8−45.9	85.9	37.9
**Percentage of SL**								
Predorsal length	42.7	39.4−48.5	43.8	1.9	39.5	39.1−42.7	40.9	1.2
Head length	34.6	30.2−40.2	34.9	1.9	33.6	31.9−35.8	33.9	0.9
Head-dorsal length	8.0	7.1−11.0	9.3	1.0	7.5	5.3−8.2	6.9	0.7
Cleithral width	33.4	31.2−38.6	33.5	1.7	35.8	30.9−35.8	32.8	1.4
Head-pectoral length	29.1	24.5−33.6	29.6	2.0	31.7	21.9−31.7	28.3	1.8
Thorax length	22.8	19.6−25.6	22.2	1.6	20.1	20.1−25.5	22.7	1.5
Pectoral spine length	30.0	22.3−29.7	27.7	1.7	36.2	27.8−36.7	31.6	2.5
Abdominal length	24.2	20.3−30.4	24.3	2.2	20.5	20.5−26.0	23.9	1.4
Pelvic spine length	25.9	20.3−29.8	23.7	2.0	27.0	23.8−27.4	25.9	1.0
Postanal length	32.3	25.9−35.9	31.3	2.5	29.2	29.2−35.3	32.8	1.6
Anal-fin spine length	9.6	5.4−12.9	10.1	1.7	16.6	7.8−16.6	10.1	1.9
Dorsal-pectoral depth	26.4	20.2−29.4	25.9	1.9	24.0	23.3−26.5	24.8	0.9
Dorsal spine length	24.3	17.7−29.2	23.0	2.1	22.5	22.5−32.7	20.0	2.2
Dorsal-pelvic depth	22.4	15.2−26.7	21.2	2.3	19.3	17.2−26.5	20.1	2.0
Dorsal-fin base length	28.1	20.4−28.1	26.0	1.7	29.9	24.9−30.6	27.4	1.6
Dorsal-adipose distance	14.3	9.0−14.3	12.0	1.9	13.3	13.1−17.4	15.4	1.2
Adipose-spine length	10.1	6.3−16.9	9.1	2.4	8.6	7.7−10.3	8.6	0.6
Dorsal adipose-caudal distance	16.0	13.4−22.0	16.3	2.3	12.1	12.1−16.5	15.0	1.0
Caudal peduncle depth	11.3	10.0−16.7	11.0	1.5	10.6	9.1−11.0	10.2	0.5
Ventral adipose-caudal distance	22.6	20.3−25.6	22.2	1.2	19.5	19.5−22.9	21.3	1.0
Adipose-anal distance	18.9	16.9−24.8	19.6	1.9	18.8	16.9−19.9	18.8	0.8
Dorsal-anal distance	33.1	29.3−35.4	32.8	1.4	12.6	12.1−19.1	13.3	1.5
Pelvic-dorsal distance	27.6	17.4−27.6	21.1	1.8	28.2	18.3−29.4	25.7	2.7
**Percentage of HL**								
Head-eye length	26.4	25.8−31.4	28.8	1.5	27.7	25.9−33.1	29.2	1.8
Orbital diameter	13.8	12.7−20.3	15.5	1.7	13.1	13.1−19.9	16.8	1.7
Snout length	67.4	62.3−69.3	65.4	1.6	69.5	56.6−72.4	62.2	4.5
Internares width	14.8	13.2−18.7	15.8	1.3	15.5	11.9−16.3	14.5	1.2
Minimal interorbital distance	30.6	27.4−35.7	29.1	1.9	31.9	24.0−32.6	28.2	2.4
Mouth length	48.8	48.8−62.3	57.5	2.5	49.7	39.8−51.9	45.9	3.5
Barbel length	10.9	4.2−10.9	8.0	1.6	5.5	4.6−8.7	7.2	1.3
Dentary tooth cup length	20.8	15.4−24.6	20.5	2.6	20.1	16.1−22.4	19.8	1.7
Premaxillary tooth cup length	19.5	16.5−25.6	19.5	2.2	18.1	17.8−24.3	20.5	1.9
Head depth	64.2	59.7−65.7	62.4	1.8	64.3	56.6−66.2	62.6	2.4

Body plates and cleithrum with minute odontodes. Odontodes slightly hypertrophied on pectoral-fin spines, gradually larger towards tips. Numerous yellowish hypertrophied odontodes along lateral margins of head including snout; odontodes small on tip of snout increasing gradually in length from anterolateral margin of snout to cheeks; longest odontodes on posteriormost portion of non-evertible cheek plates. Eye small (orbital diameter 13−20% HL), dorsolaterally positioned. Oral disk transversely ellipsoid. Lower lip not reaching transverse line between gill openings. Lower lip covered with numerous small papillae. Maxillary barbel developed. Mouth relatively large. Premaxillary teeth 33−70 per ramus; dentary teeth 39−74 per ramus. Teeth bifid, medial cusp large and rounded, lateral cusp minute and rounded. Jaws wide, dentaries forming oblique angle, premaxillaries almost co-linear.

Dorsal fin I,7, origin approximately at midpoint between pectoral- and pelvic-fin origins, last dorsal-fin ray reaching adipose fin when depressed. Pectoral fin I,6, spine tip slightly curved inward, covered with enlarged odontodes distally; depressed tip reaching one-third length of pelvic-fin spine. Pelvic fin I,5, spine tip curved inward, almost reaching anal-fin origin when depressed. Anal fin I,5, spine tip straight, reaching sixth plate posterior to its origin. Caudal fin I,7-7I, distal margin concave, inferior lobe longer than superior. Adipose fin with straight spine, preceded by single median preadipose plate.

#### Color in alcohol.

Ground color dark brown on back and sides of body, and lighter brown ventrally. Anterior portion of head to posterior margin of orbits with many small, crowded, white spots; spots getting abruptly larger on posterior portion of head, continuing on body, fading along posterior portion of dorsal fin and forming mottled pattern. Dorsal-fin spine rays and membranes with 6−7 dark bars. Pectoral, pelvic, anal with 4−5 dark bars and caudal-fin with four dark bars. Hypertrophied odontodes along head margin yellowish.

#### Sexual dimorphism.

Males possess a papilla posterior to urogenital opening, an attribute absent in females. Both sexes in *Pseudancistrus kayabi* exhibit highly hypertrophied odontodes along snout margin, as well as in other species of *Pseudancistrus* (Armbruster 2004b).

#### Etymology.

The specific name “kayabi” is a reference to the Kayabi indigenous people that inhabited the region of the rivers Arinos, dos Peixes and Teles Pires, in Mato Grosso State, Brazil. A noun in apposition.

#### Distribution.

**Figure 2.**
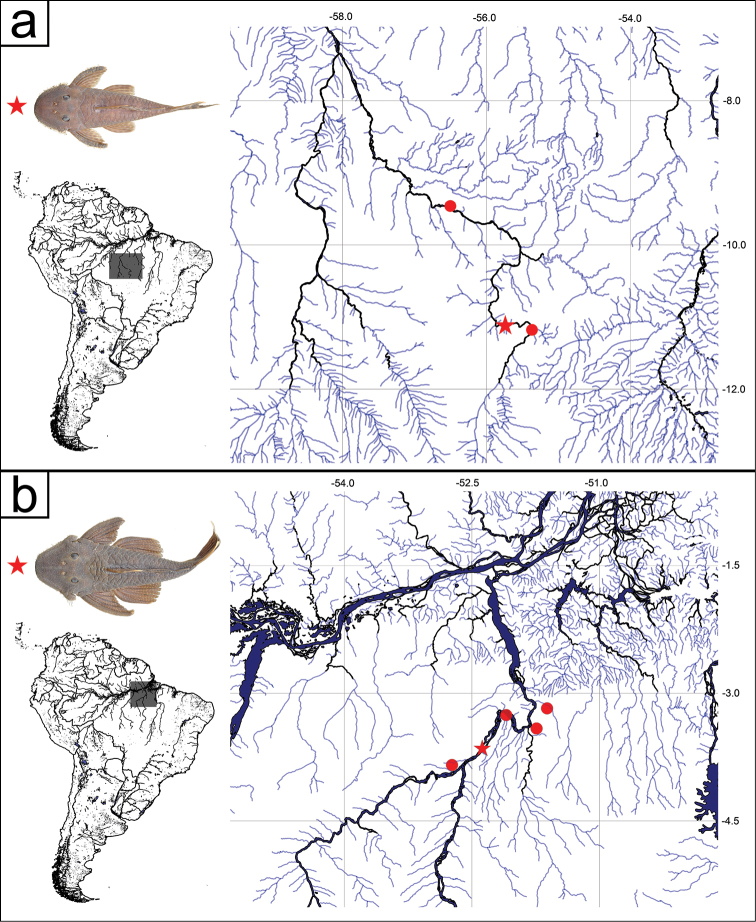
Distribution maps. **a** *Pseudancistrus kayabi*. Star shows holotype locality, rio Teles Pires, 10°58'30"S, 55°44'03"W. Circle shows paratype locality **b** *Pseudancistrus asurini*. Star shows holotype locality, rio Xingu, 03°39'05"S, 52°22'42"W. Circles show paratypes localities.

*Pseudancistrus kayabi* is known from the rio Teles Pires, rio Tapajós basin, municipality of Itaúba and Paranaíta, Mato Grosso State, Brazil (Fig. 2a).

### Pseudancistrus asurini sp. n.

Taxon classificationAnimaliaSiluriformesLoricariidae

http://zoobank.org/02D58958-6DD0-441A-8755-96F8387F2C33

**Figure 3.**
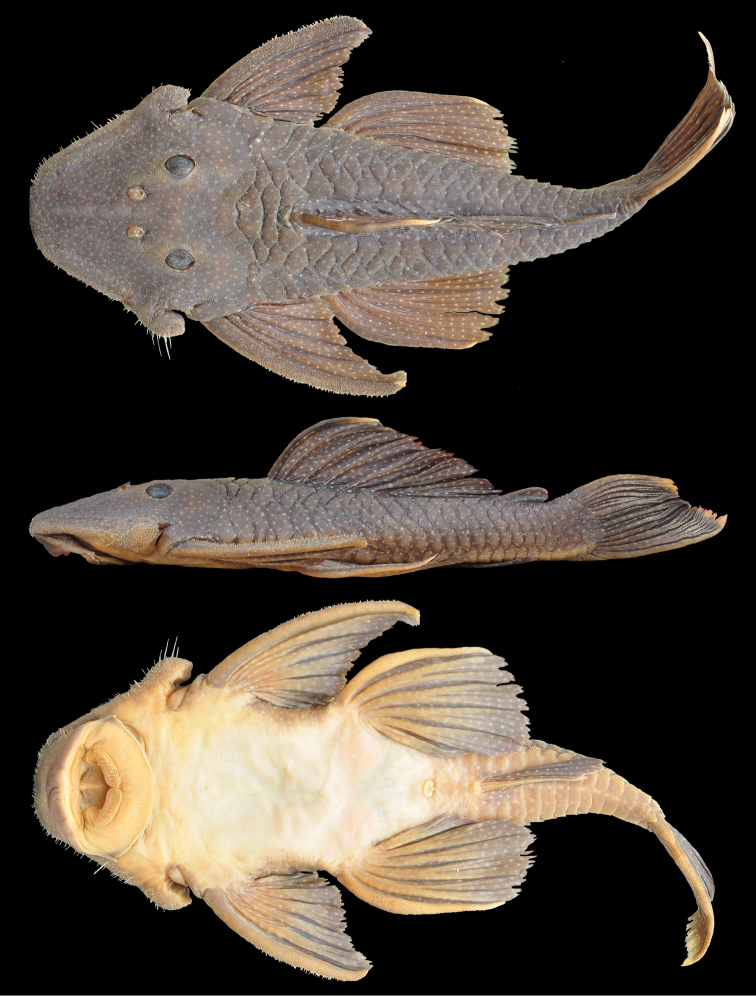
*Pseudancistrus asurini*, holotype, MZUSP 116323, male 195.8 mm SL, from rio Xingu (Amazon basin), municipality of Altamira, Pará State, Brazil.

**Figure 4.**
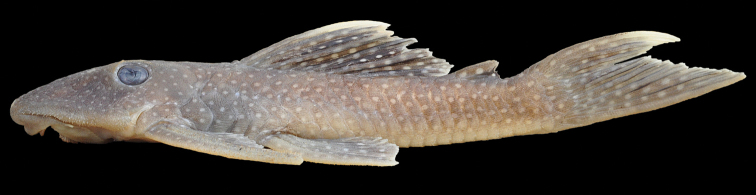
*Pseudancistrus asurini*, paratype, LBP 16551, female 100.5 mm SL, from rio Xingu (Amazon basin), showing the dorsal and caudal fins tips whitish.

Figures 3 , 4 , Table 1

Pseudancistrus sp. L17: Covain and Fisch-Muller 2012: 232−233 (Table 1), 235 (Fig. 2), 237 (Fig. 3), 242 (Fig. 4). – Silva et al. 2014: 6 (Fig. 2), 14 (Fig. 6), 16 (Table 3), 17 (Fig. 7).
Pseudancistrus sp. L67: Lujan et al. 2015: 281 (Fig. 3).

#### Holotype.

MZUSP 116323, male, 195.8 mm SL. Brazil: Pará State: municipality of Altamira: rio Xingu (Amazon basin), Cachoeira do Espelho, 03°39'05"S, 52°22'42"W, 18 November 2011, OT Oyakawa, JLO Birindelli, C Moreira, A Akama, LMS Souza.

#### Paratypes.

All from Brazil: Pará State: municipality of Altamira: Amazon basin. AUM 65640, 2, 79.1−82.9 mm SL, rio Xingu, Cachoeira da Mucucura, 03°24'31"S, 51°44'40"W, 09 November 2011, OT Oyakawa, JLO Birindelli, C Moreira, LMS Souza. LBP 16551, 2, 75.6−101.4 mm SL, rio Xingu, 03°15'24"S, 52°05'47"W, 28 September 2012, C Oliveira, R Britzke, LMS Sousa. MZUSP 107174, 4, 45.9−123.4 mm SL, rio Xingu, Cachoeira de Mucura, 03°24'51"S, 51°44'23"W, ECIX team. MZUSP 107179, 2, 62.3−68.7 mm SL, rio Xingu, Cachoeira do Mucura, 03°24'51"S, 51°44'23"W, 07 July 2010, ECIX team. MZUSP 107435, 3, 74.6−105 mm SL, rio Xingu, 03°10'40"S, 51°36'58"W, 26 September 2007, FCT Lima, AK Zeinad. MZUSP 111285, 2, 84.4−106.6 mm SL, rio Iriri (trib. rio Xingu) Cachoeira Grande, 03°50'37"S, 52°44'02"W, OT Oyakawa, JLO Birindelli, C Moreira, A Akama, LMS Souza. MZUSP 111441, 6, 49.5−152.3 mm SL, rio Xingu, Cachoeira da Mucucura, 03°24'31"S, 51°44'40"W, 09 November 2011, OT Oyakawa, JLO Birindelli, C Moreira, LMS Souza. MZUSP 111558, 1, 91.4 mm SL, collected with holotype.

#### Diagnosis.

The new species differs from all congeners by having the dorsal-and caudal-fin tips whitish (Fig. 4) (vs. entirely dark). It further differs from *Pseudancistrus reus* and *Pseudancistrus kayabi* by having conspicuous whitish spots on the body (vs. body mottled or with bars in *Pseudancistrus reus* and with whitish spots that fade along the body and can cover more than one plate in *Pseudancistrus kayabi*). It is also distinguishable from *Pseudancistrus depressus* and *Pseudancistrus barbatus* by having the snout with yellowish odontodes (vs. reddish-brown) (see Fig. 3 in De Chambrier and Montoya-Burgos 2008 for comparison) and from *Pseudancistrus nigrescens*, *Pseudancistrus corantijniensis*, and *Pseudancistrus zawadzkii* by having smaller whitish spots covering the body which increase gradually in size on the head (diameter 0.3−0.8 mm) and further on the body (diameter 0.7−1.3) (vs. spots abruptly increasing size between the head (diameter 1.1−1.3) and the body (diameter 2.6−2.3 mm). In addition, the new species is distinguished by a shorter predorsal length, 39−43% SL (vs. 43−46% in *Pseudancistrus zawadzkii* and 43−45% in *Pseudancistrus nigrescens*), a smaller dorsal pectoral depth, 23−27% SL (vs. 27−31% in *Pseudancistrus zawadzkii*); a smaller caudal peduncle depth, 9−11% SL (vs. 13−14% in *Pseudancistrus zawadzkii* and 13% in *Pseudancistrus nigrescens*), a shorter barbel, 5−9% HL (vs. 10−11 in *Pseudancistrus nigrescens*), and head depth, 57−66% SL, which is smaller than in *Pseudancistrus zawadzkii* (67−73%) but greater than in *Pseudancistrus barbatus* (41−53%).

#### Description.

Morphometric data is presented in Table 1. In lateral view, dorsal profile convex from snout tip to dorsal-fin origin; straight, gradually descending from dorsal-fin origin to posterior insertion of adipose fin; straight, steeply ascending to insertion of caudal fin; ventral profile flat from snout tip to anal-fin origin; shallowly concave from anal-fin insertion to lower caudal-fin spine; greatest body depth at dorsal-fin origin. In dorsal view, greatest body width across cleithral region; snout broadly elliptical; body decreasing in width from opercular region to caudal fin. Cross-section of body between pectoral and pelvic fins rounded dorsally and flattened ventrally; cross-section of caudal peduncle ellipsoid.

Body almost entirely covered with plates, except on ventral portions of head, abdomen, and dorsal-fin base. Five lateral rows of dermal plates, dorsal plates 21−22, lateral mid-dorsal plates 18−22, lateral median plates 22−23, lateral mid-ventral plates 23−24, lateral ventral plates 18−19. Three predorsal plates; seven plates below dorsal-fin base; four plates between dorsal fin and adipose fin; five rows of plates on caudal peduncle. Dorsal spinelet present.

Body plates and cleithrum with minute odontodes. Odontodes gradually getting larger towards tips on pectoral-fin spines. Numerous whitish hypertrophied odontodes along lateral margins of head including snout; homogenous in length excepting in anterior portion of snout where odontodes are smaller; longest odontodes on posteriormost portion of non-evertible cheek plates. Eye small (orbital diameter 13−10% HL), dorsolaterally positioned. Oral disk transversely ellipsoid. Lower lip not reaching transverse line between gill openings. Lower lip covered with numerous small papillae. Maxillary barbel poorly developed. Mouth relatively large. Premaxillary teeth 38−77 per ramus; dentary teeth 39−86 per ramus. Teeth bifid, medial cusp large and rounded, lateral cusp minute and rounded. Jaws wide, dentaries forming oblique angle, premaxillaries almost co-linear.

Dorsal fin II,7, origin approximately at midpoint between pectoral- and pelvic-fin origins, last dorsal-fin ray not reaching adipose-fin when depressed. Pectoral fin I,6, spine tip not curved inward; depressed tip reaching one-third length of pelvic-fin spine. Pelvic fin I,5, spine tip curved inward, almost reaching anal-fin origin when depressed. Anal-fin I,5, spine tip straight, reaching fifth plate posterior to its origin. Caudal fin I,7-7I, distal margin concave, inferior lobe longer than superior. Adipose fin with almost straight spine, preceded by single median preadipose plate.

#### Color in alcohol.

Ground color dark brown on back and sides of body, and lighter brown ventrally. Anterior portion of head to posterior margin of orbits with many small, crowded, white spots; spots increasing slightly and gradually in size between snout to body. Dorsal plate series usually with two or three spots per plate in anterior portion of body and one spot on posterior portion of body. Mid-dorsal plates usually with two or three spots per plate. Lateral median plates with one or two spot per plate. Mid-ventral plates and ventral plates with two or three spots per plate. Dorsal-fin spine, rays and membranes with small round spots. Adipose fin with three small spots on spine and membrane. Pectoral, pelvic, anal and caudal fins with numerous and white spots of equal size. Dorsal and caudal fin tips whitish. Hypertrophied odontodes along head margin yellowish.

#### Color in life.

Similar to pattern described for alcohol individuals, but with ground color dark greenish-brown, and with yellow spots on body and on tips of dorsal and caudal fins.

#### Sexual dimorphism.

Males possess a papilla posterior to urogenital opening, an attribute absent in females. Both sexes in *Pseudancistrus asurini* exhibit highly hypertrophied odontodes along snout margin, as well as in others species of *Pseudancistrus* (Armbruster 2004b).

#### Etymology.

The specific name “asurini” is a reference to the Asurini indigenous peoples who inhabit the right margin and median portions of rio Xingu, close to the municipality of Altamira in Pará State, Brazil. A noun in apposition.

#### Distribution.

*Pseudancistrus asurini* is known from the rio Xingu, municipality of Altamira, from the Xingu river basin, Pará State, Brazil (Fig. 2b).

## Discussion

The two new species, *Pseudancistrus kayabi* and *Pseudancistrus asurini*, are typical *Pseudancistrus* (sensu Chambrier and Montoya-Burgos 2008), recognized by non-evertible cheek plates and the presence of hypertrophied odontodes along the snout margin. This last character is shared with species of *Lithoxancistrus* and *Pseudolithoxus*. However, in *Pseudancistrus*, the odontodes along the snout are quite well developed, especially in *Pseudancistrus kayabi*. Additionally, *Pseudolithoxus* (Armbruster and Provenzano 2000) can be distinguished from *Pseudancistrus* by the presence of three rows of plates on the caudal peduncle (vs. five), and *Lithoxancistrus* can be distinguished from *Pseudancistrus* by the presence of three buccal papillae (vs. one; Isbrücker et al. 1988).

The most conspicuous character used to distinguish the two new species from all other described *Pseudancistrus* is the coloration pattern. *Pseudancistrus kayabi* has a pattern of dark bars on dorsal and caudal fins (Fig. 1) as in *Pseudancistrus reus* from the Caroní River, Venezuela. However, *Pseudancistrus reus* possesses dark brown bars also on the body. This character is absent in *Pseudancistrus kayabi*, which has a dark brown base color with whitish spots fading posterior to the dorsal fin and are large enough to cover more than one lateral body plate, a pattern that is similar to that found in *Pseudancistrus nigrescens*.

*Pseudancistrus asurini* has whitish tabs on the dorsal- and caudal-fin tips (Fig. 4) in juveniles and medium-sized adults (to approximately 100 mm SL), a pattern unique among *Pseudancistrus*. This character is similar to that found in *Baryancistrus xanthellus* (Py Daniel et al. 2011) and *Baryancistrus chrysolomus* (Py Daniel et al. 2011), both of which are also from the rio Xingu basin and live sympatrically with *Pseudancistrus asurini*. Additionally, the new species *Pseudancistrus asurini* has a color pattern consisting of spots that increase in size from the head (diameter 0.3−0.8 mm) to posterior part of body (diameter 0.7−1.3). The species *Pseudancistrus nigrescens*, *Pseudancistrus corantijniensis*, and *Pseudancistrus zawadzkii* present a similar coloration pattern; however, the size of the spots increase abruptly from the head (diameter 1.1−1.3) to posterior part of body (diameter 2.6−2.3 mm).

### Comparative material examined

*Guyanancistrus brevispinis* (Heitmans, Nijssen & Isbrücker, 1983): LBP 5253, 2, 58.5–83.8 mm SL, MZUSP 103488, 23, 102.3–55.1 mm SL, Jari river, Brazil; ANSP 189128, 3, 56.8−125.7 mm SL, Marowini river, Sipalawini, Suriname. *Pseudancistrus zawadzkii* Silva, Roxo, Britzke & Oliveira, 2014: Holotype, MZUSP 115056, 116.4 mm SL, Tapajós river, Brazil; Paratypes, LBP 15045, 2, 97.9−128.7 mm SL, LBP 17724, 1, 87.5 mm SL, LBP 16195, 1, 116.4 mm SL. *Pseudancistrus barbatus* (Valenciennes, 1840): ANSP 177366, 2, 76.5−103.7 mm SL, Burro Burro river, Water Dog Falls, Essequibo river basin, Guyana; ANSP 189119, 3, 75.1−151.5 mm SL, Lawa river, Sipalawini, Suriname. *Pseudancistrus nigrescens* Eigenmann, 1912: ANSP 177379, 5, 96.4−133.5 mm SL, Burro Burro river, Water Dog Falls, Essequibo river basin, Guyana. *Lithoxancistrus orinoco* (Isbrücker, Nijssen & Cala, 1988): ANSP 160600, 6, 68.0−78.5 mm SL, Orinoco river, Venezuela. *Pseudancistrus pectegenitor* Lujan, Armbruster & Sabaj, 2007: ANSP 190755, 1, 206.2 mm SL, Ventuari river, Orinoco river basin, Venezuela. *Pseudancistrus sidereus* Armbruster, 2004b: ANSP 185321, 4, 148.6−154.1 mm SL, Casiquiari river, Venezuela.

## Supplementary Material

XML Treatment for Pseudancistrus kayabi

XML Treatment for Pseudancistrus asurini
